# Stiffness Hardening Effect of Wire Rope Isolators under Small Cyclic Loads for Vibration Isolation

**DOI:** 10.3390/ma17204983

**Published:** 2024-10-11

**Authors:** Mingyang Fu, Zhenyu Yang

**Affiliations:** 1 Key Laboratory of Earthquake Engineering and Engineering Vibration, Institute of Engineering Mechanics, China Earthquake Administration, Harbin 150000, China; 2112216233@e.gzhu.edu.cn; 2 Earthquake Engineering Research & Test Center, Guangzhou University, Guangzhou 510006, China

**Keywords:** wire rope isolator, mechanical property, stiffness increase, vibration isolation

## Abstract

Wire rope isolator (WRI) devices are widely used in vibration reduction industrial equipment, and stiffness is the key parameter that determines isolation effectiveness. WRI devices show slight nonlinearity under small loads, and the manufacturers generally only provide the initial parameters. To investigate the mechanical behavior changes in the WRI devices under repeated loads, five types of WRI specimens were tested under various amplitudes, loading speeds, and preloads. The test results of large symmetrical compression and tension loads showed that the WRI devices demonstrated stable hysteresis curves under repeated loads, while the hysteresis curves were independent of the loading speed. The test results of small cyclic loads with large preloads show that the stiffness of the WRI specimen follows the logarithmic law, with the cycle number under various loading conditions. Particularly, the stiffness of the specimen increases by about 10–30% after 50 cycles. The initial stiffness *K*_a_ decreases linearly with the preloads, while the decrease is quadratic in relation to the cyclic load. The hardening coefficient *C*_a_ shows a positive correlation with the loading capacity of the WRI devices, while it shows a negative correlation with the preload and cyclic load amplitudes. It is recommended to consider the stiffness increase in the WRI devices during the evaluation of isolation effectiveness.

## 1. Introduction

Wire rope isolator (WRI) devices are widely used in vibration reduction industrial equipment; examples include the electric switchboards on naval ships [1], accurate manufacturing applications [2], and stay cables in bridges [3]. In addition, WRIs can also be used in the seismic protection of a wide range of structures, including high-voltage electrical equipment [4], equipment in buildings [5,6], and computer cabinets [7]. As the isolated structure may generate excessive displacement under severe earthquakes, the wire rope can also serve as a restrainer of the isolation layer [8]. Before the design of a WRI platform, the WRI device is tested using quasi-static loads, and the hysteresis curves, as well as other mechanical properties, are obtained. Meanwhile, the real-world structure generally shows uncertainties and changes in the seismic inputs, dimensions, and mechanical properties [9]; these need to be considered in the analyses of the isolated structures. For example, when the mechanical properties change due to uncertainties, they can affect the seismic responses of structures [10,11].

Vibration frequency is the key parameter involved in vibration isolation [12,13], while the stiffness of structural components may change during long-term service or cyclic loads. The steel material shows stiffness hardening under repeated fatigue loads, and the stiffness increases significantly after 10^5^ cycles [14]. Moreover, another low-yield-point steel shows cyclic hardening and softening behavior [15]. In addition, the rubber material, which is used in seismic isolators, shows stiffness hardening in low temperatures [16]. Particularly, it should be pointed out that property changes in isolators due to variations in the temperature result in ±10% changes in reliability [17]. According to recent studies, the mechanical properties of the seismic and vibration isolators show considerable changes during long-term service; an example of this would be the widely used rubber bearings [18,19].

The hysteresis behavior of WRIs comes from the friction between wires, and is generally described using the Bouc–Wen model [20], and the modified version of the Bouc–Wen model is needed if the displacement of WRIs is large [21]. Particularly, the asymmetric Bouc–Wen model is preferred to describe the tension–compression behavior of WRIs [22]. Meanwhile, the tests show that WRIs still exhibit symmetric behavior under tension and compression loads if the displacement is small [23]. In addition to the popular Bouc–Wen model, the hysteresis behavior of WRIs can also be described using the artificial neural network method [24]. Alternatively, the Vaiana–Rosati model is also proposed for modeling the axial behavior of WRIs [25].

Although the isolation is designed based on specified parameters, the actual mechanical behavior of the isolators in the real world may still change. Therefore, the isolator specimens were tested many times to obtain the probabilistic distribution of the key mechanical properties. Based on tests of 244 rubber bearings, a popular seismic isolator for buildings and bridges, the equivalent shear stiffness was found to follow the I-type minimum-value distribution under repeated loads [26]. In addition, another test, based on 38 rubber bearings, showed that the ultimate horizontal deformation followed the normal distribution [27]. Moreover, a sensitivity analysis showed that variation in the diameter of the lead core significantly affected the mechanical behavior of the lead rubber bearing (LRB) [28]. In another test, repeated loading tests performed on the rubber bearings showed that the mechanical properties followed a lognormal or general extreme distribution [29]. The obtained distribution parameters, given above, can be adopted in the seismic analysis. For example, the stiffness and yield force of isolators are assumed to follow a uniform distribution [30]. As there is little literature related to the parameter variation in WRIs, studies on the rubber bearings can provide references for those on WRIs.

Existing studies concentrate on the probabilistic distribution of the mechanical properties of rubber isolators. Meanwhile, WRIs, another popular seismic or vibration isolator used in industrial facilities, also show variations in key mechanical properties under repeated loads. Since WRIs generally experience several or hundreds of cycles due to earthquakes or vibrations, the mechanical property changes in WRI devices under repeated loads need further investigation.

To obtain the cyclic mechanical property change law of WRIs, in this study, several WRI devices were tested many times on a universal testing machine, and the hysteresis curves were obtained. Then, the equivalent stiffness in various scenarios was identified and collected. Finally, the stiffness change law of WRI devices under repeated loads was derived.

## 2. Materials and Methods

### 2.1. Test Specimen

**Figure 1:**
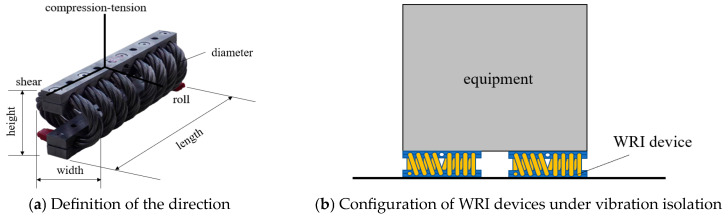
The test specimen of the WRI specimen.

To obtain the mechanical behavior of WRI devices, five types of WRI device produced by the Anlijing company (Baoding, China) were used in a quasi-static test. The WRI devices came from this manufacturer. The WRI device was composed of rolled wire rope cables and crimped mount bars. It was installed via holes in the bars, as shown in Figure 1a. Generally, WRIs have eight cycles, which are arranged in a series. In addition, the WRI device used in this study also had eight loops. The WRI device was deformable in three directions, namely the roll, shear, and compression–tension (C-T) directions. In rocking isolation, the WRI devices were installed vertically, as shown in Figure 1b, and the superstructure was generally a slender structure. In addition, in the vibration isolation, the superstructure always stood on the WRI devices. Therefore, the WRI device was primarily subjected to tension and compression forces, as the deformation in roll and shear directions were generally small. In the seismic analysis, when rocking isolation was used, the WRI could be simplified as a vertical axial damper [4]. Therefore, in this study, only the mechanical properties in the C-T direction were considered.

Table 1 lists the physical and mechanical properties of the five types of WRI specimen. The definitions of the length, width, height and diameter follow those used in Figure 1a, while the data come from the manufacturer. Notably, as the WRI is generally used for vibration isolation, the vibration and shock stiffness correspond to vibration mitigation under steady-state and shock loads, respectively.

### 2.2. Test Facility and Method

**Figure 2:**
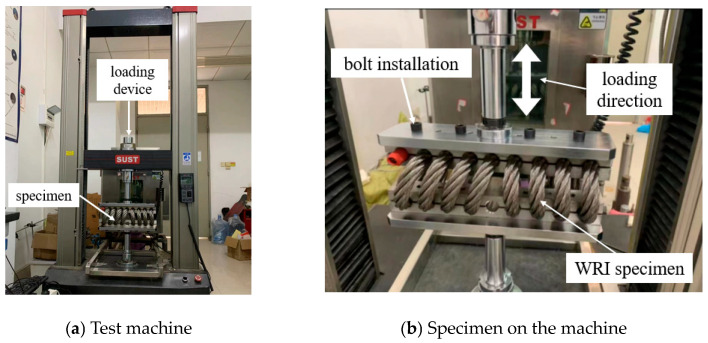
Photo and components of the test machine.

Since only the C-T load is considered, a universal testing machine was employed to perform the test, as shown in Figure 2a. The loading capacity of the machine was 50 kN in tension or compression situations, and the machine could only load the specimen in the uniaxial direction using quasi-static loads. As the specimen was subjected to compression as well as tension loads, a connection component was used to connect the specimen and the test machine, as shown in Figure 2b.

When the WRI devices are installed on the structure, they are subjected to repeated loads from several seismic events during their service lives. Otherwise, if the WRI devices are used for vibration isolation, they also experience repeated small loads. Therefore, the specimen was subjected to repeated loads to investigate the changes in some key properties.

The five specimens were subjected to cyclic loads for many cycles; Table 2 lists the deformation, loading speed, and cycle number in various scenarios. The test consisted of five programs, given as T1 to T5. In the first test T1, the first specimen ALJ-8110133 (S2), which had the largest loading capacity among the five specimens, was used. The S2 specimen was loaded with various speeds, loads, and cycle numbers to illustrate the dependency of the mechanical behaviors. Then, in the second test, T2, the S1 specimen was subjected to small loads, and we used only 20% of the static capacity (STC) multiple times to demonstrate the mechanical properties of the specimen under repeated loads. In the third test, T3, the S1 specimen was loaded using increasing loads from 20% to 80% of the STC to investigate the mechanical property changes. Finally, in the fourth test T4, several specimens—S2, S3, S4, and S5—were loaded under small cyclic loads to identity the variation between different types of specimen.

During the test, the specimens were first subjected to the preload; then, the load was varied following the sine wave with the specified amplitudes. If the preload was zero, then the specimens were subjected to the sine loads directly. In addition, we recorded the time history of axial deformation and reaction forces using built-in sensors, which were already accurate enough. According to the manufacturer of the test machine, the error in the built-in force and displacement sensor is within ±0.5% of the test values.

## 3. Test Results

### 3.1. Mechanical Behaviors of the WRI Specimen under Large Repeated Loads

The S2 specimen was loaded with displacements of 2.0, 5.0, 10.0, 12.5, 15.0 and 20.0 mm, respectively. When the displacement was only 2.0 mm, the specimen had little visible deformation. Meanwhile, when the displacement reached 20.0 mm, the wire ropes had significant lateral deformation, and the specimen was supposed to experience slight geometric nonlinearity.

**Figure 3:**
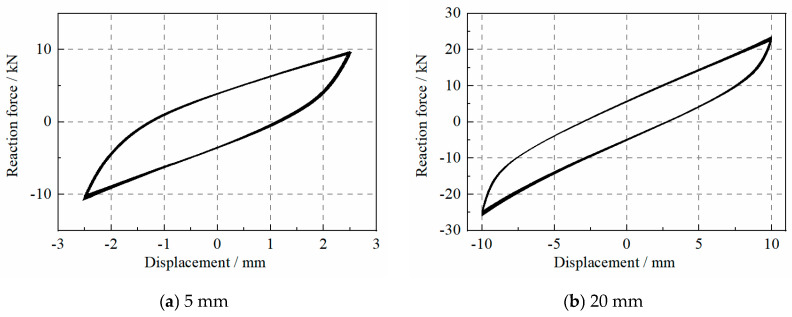
The force–deformation curve of the WRI specimen under repeated loads.

The displacement and reaction forces of the specimen under various levels of displacement were recorded, as shown in Figure 3. The S2 specimen showed stable behaviors under the cyclic loads when the displacement was small or large.

The allowable vertical static load of the S2 specimen is 12.28 kN, and the corresponding displacement is only around 5.2 mm. In real-world vibration isolation involving WRI devices, the vertical displacement is generally small and is supposed to be smaller than 1.0 mm. Therefore, in this study, as the WRI devices were used for vibration isolation, the asymmetric behavior of the WRI device in the C-T direction was ignored.

**Figure 4:**
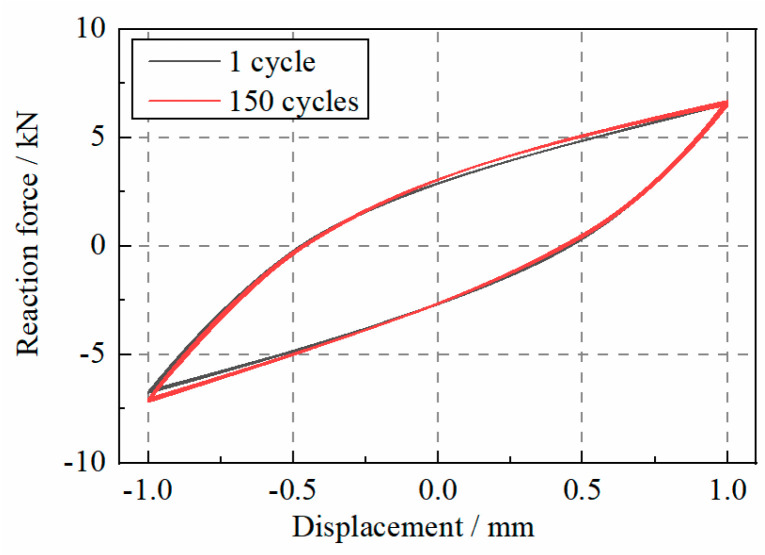
The force–deformation curve of the WRI specimen at the first and last cycles.

As listed in Table 2, the WRI specimen experienced many cycles under repeated loads. As shown in Figure 4, after 150 cycles of loads from T1-S2-D1 to T1-S2-D3, the specimen still showed the same behavior as it did during the first cycles. The maximum force had an error of only 1.1%, while the minimum force’s error was 5.8%. Therefore, the WRI specimen could sustain its mechanical behavior under hundreds of cyclic loads.

**Figure 5:**
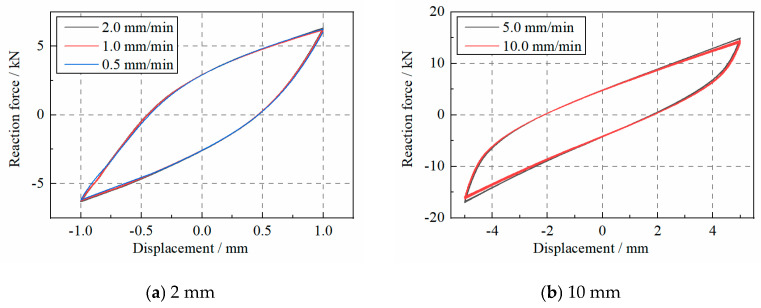
The force–deformation curve of WRI specimen under various loading speeds.

WRI devices dissipate the input energy via the friction behavior between wires, and Figure 5 shows the hysteresis curves of the specimen under various loading speeds. When the displacement is 2.0 mm, the curves of the specimen under 0.5, 1.0, and 2.0 mm/min nearly overlap. Moreover, when the displacement is 10.0 mm, the loading speeds of 5.0 and 10.0 mm/min also have little effect on the hysteresis curves. Therefore, the mechanical behavior of the WRI specimen is independent of the small loading speeds. In other words, a larger loading speed of the specimen during the test is preferred to minimize the time consumed.

### 3.2. Mechanical Properties of the WRI Specimen under Small Cyclic Loads

The above tests employ large cyclic loads, which simulate the load on the WRI devices during seismic events. When the WRI devices serve as vibration isolators, the load is much smaller, and so the corresponding load configuration is also considered. Since the specimen showed stable behaviors under various loading conditions, demonstrating particularly that the force–deformation curves were independent of the loading speed, all the specimens were loaded under 10 mm/min to save the time.

**Figure 6:**
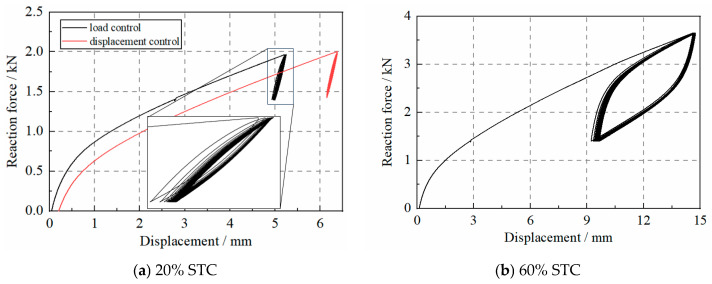
The force–deformation curve of WRI specimens under small loads.

**Figure 7:**
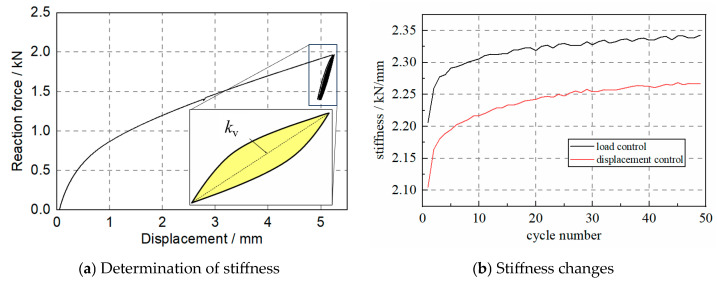
The axial stiffness of the WRI specimen.

Figure 6 shows the force–displacement curves of the WRI specimen under small, repeated loads. The specimen was first subjected to the dead load of the superstructure and was then loaded using small amplitudes. In T2-S1-F1, the load amplitude was 20% STC, and the specimen was loaded for 50 cycles. As shown in Figure 7a, the force–displacement curves of the 50 cycles seem not to overlap, which indicates that the stiffness of the specimen varies in the 50 cycles. In addition, in T2-S2-D6, in which the loading procedure was controlled by the displacement, changes in stiffness also occurred. Then, when the loading amplitude increased to 80% STC, as shown in Figure 7b, the specimen showed a similar phenomenon in that the force–displacement curves changed over cycles.

Since the stiffness is the key parameter in terms of the effect of isolation, the change in stiffness may affect isolation’s effectiveness.

As the hysteresis curves of the specimen in this test were recorded, the test values of the specimen, as shown in Figure 7a, were calculated as follows:

(1) $$K_{v}=\frac{F_{2}-F_{1}}{X_{2}-X_{1}}$$

where *F*_1_ and *F*_2_ are the maximum and minimum force in the cycle, and *X*_2_ and *X*_1_ are the corresponding vertical deformations, respectively. The test data were divided using Python code in several loops, and the stiffness of each loop was estimated using Equation (1).

Figure 7b shows the changes in the stiffness of the S1 specimen under various loading cycles. The stiffness of the specimen increases with the cycle number, and the stiffness after 50 cycles is over 10% larger than the initial one. In addition, the specimen shows stiffness increases in both the load and displacement control loading protocols.

**Figure 8:**
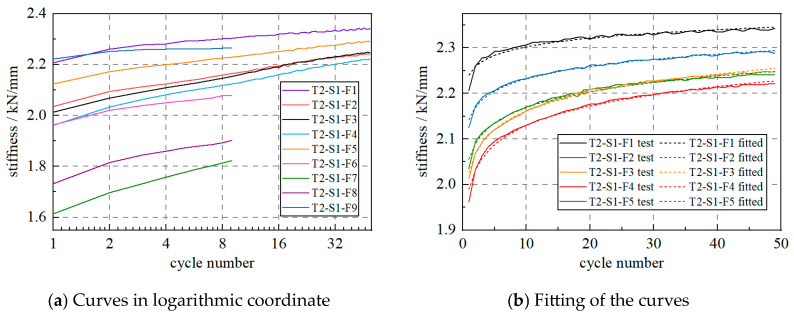
WRI device stiffness changes in various scenarios.

The WRI specimen was loaded 9 times, using the same loading configuration, to further investigate the stiffness increase effect. As shown in Figure 8a, the specimens all showed stiffness increases with the cycle number. In addition, when the logarithmic coordinate was used, the curves became straight lines for all the test scenarios. This indicates that the relationship between the cycle number and stiffness is as follows:

(2) $$K_{v}=C_{a}\ln\left(N\right)+K_{a}$$

where *C*_a_ is the hardening coefficient, *N* is the cycle number, and *K*_a_ is the initial stiffness. The hardening coefficient and initial stiffness can be identified using the least-squares method, which is performed using Python codes, and Figure 8b shows the test and fitted curves. The logarithmic function can accurately predict the stiffness change effect of the WRI specimen.

**Figure 9:**
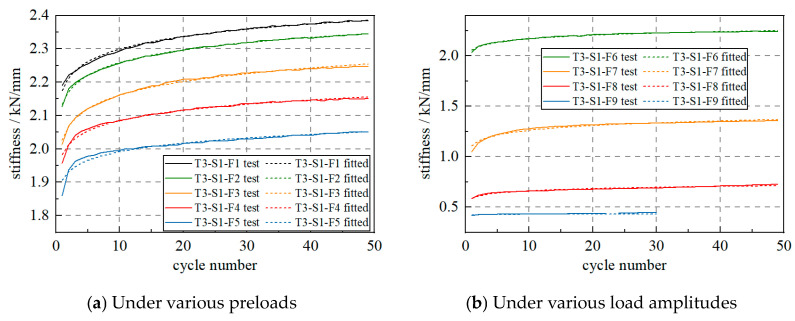
WRI device stiffness changes under various preloads and load amplitudes.

To investigate the influence of the load, the S1 specimen was loaded using various preloads and amplitudes. As shown in Figure 9a, the S1 specimen was subjected to preloads of 20%, 35%, 50%, 65% and 80% in scenarios from T3-S1-F1 to T3-S1-F5, respectively. Under various preloads, the specimen showed similar stiffness change laws, which could be described using the logarithmic function. Then, as shown in Figure 9b, the S1 specimen was subjected to various load amplitudes of 20%, 40% 60% and 80% of the STC. Similarly, the specimen still showed logarithmic increases in stiffness with the cycle number.

**Figure 10:**
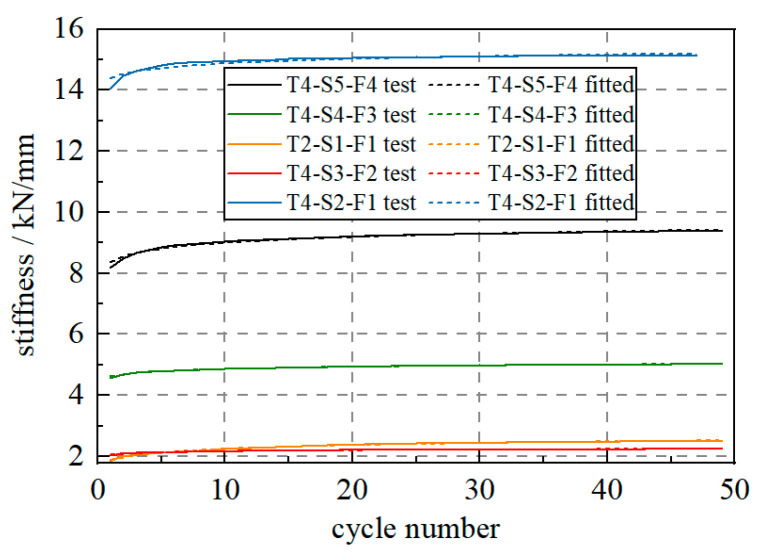
WRI device stiffness changes for various WRI devices.

The above tests all employ the S1 specimen; then, the other four specimens are also used to further verify the stiffness change law. As shown in Figure 10, all the five specimens are subjected to 20% STC loads, and all the five specimens follow the logarithmic law in terms of stiffness changes.

## 4. Discussion

During the repeated load test with large amplitudes, the WRI specimen shows stable hysteresis curves under cyclic loads. In addition, the hysteresis curves are independent of the loading speed. Meanwhile, the stiffness of the WRI specimen shows a probabilistic distribution under different loading scenarios, in which the specimen is released from the test facility after each test. More important, the stiffness of the specimen keeps increasing as the load cycles.

The test results show that the stiffness of the WRI specimen follows the logarithmic law with the cycle number under various loading conditions. Particularly, the stiffness of the specimen increases by about 10–30% after 50 cycles, which may affect vibration isolation’s effectiveness. Among the tests in Table 2, the stiffness increase in T1 test, in which the S2 specimen is subjected to 20% STC cyclic loads, is around 10% after 50 cycles. In T2 tests, although the preload varies from 20% to 80% STC, the stiffness increase stays around 10% after 50 cycles. Meanwhile, when the load is 40% and 60% STC, the stiffness increase reaches around 25%, which is much larger than that of the other configurations. Additionally, among the five types of specimens, the ALJ-819127 specimen shows the maximum stiffness increase, up to 33% after 50 cycles, while other specimens see increases below 15%. Overall, the stiffness increase of the WR devices is typically around 15%.

**Figure 11:**
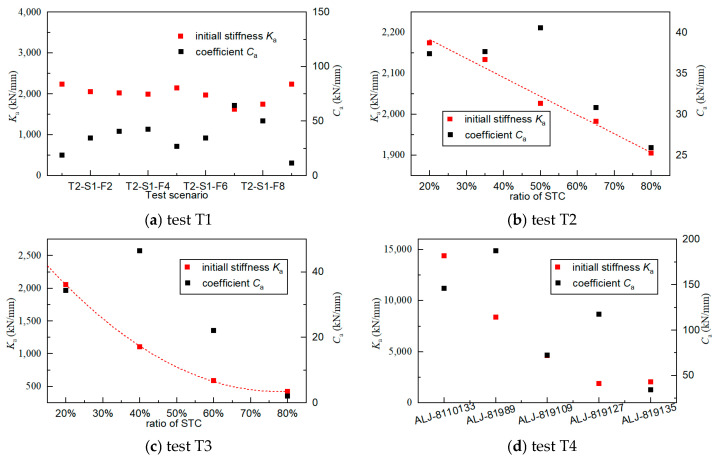
The fitted coefficient of stiffness change curves.

The coefficient *C*_a_ and initial stiffness *K*_a_ are identified using Equation (2), and the results in the four tests are shown in Figure 11. The standard deviation σ of the result is defined as follows:

(3) $$\sigma =\sqrt{\frac{\sum_{i}^{N}\left(x_{i}-\bar{x}\right)}{N}}$$

where *x*_i_ is the *i*th result, $\bar{x\;}$ is the averaged value, and *N* is the number of the result. When the S2 specimen is subjected to nine tests, the initial stiffness *K*_a_ remains stable at 2000 kN/mm, with a standard deviation of 205 kN/mm. Meanwhile, the coefficient *C*_a_ is 35.90 kN/mm on average, with a standard deviation of 15.98 kN/mm.

When the preload and cyclic load vary, the initial stiffness *K*_a_ changes accordingly. The initial stiffness *K*_a_ decreases linearly with the preloads, while is quadratically related to the cyclic load, as shown by the red dotted lines in Figure 11b,c, respectively. Meanwhile, the coefficient *C*_a_ tends to decrease with the preload and cyclic load, although there are exceptions in some cases. Particularly, the coefficient *C*_a_ decreases sharply with the cyclic load; when the cyclic load reaches 80% STC, *C*_a_ is only 2.08 kN/mm.

In this study, five types of specimen are used. As shown in Figure 11d, the initial stiffness *K*_a_ decreases with the loading capability, which is listed in Table 2. Additionally, the coefficient *C*_a_ tends to decrease with the loading capability. The ALJ-819135 (S1) has a larger *K*_a_, while the ALJ-819127 has a larger *C*_a_, and so the ALJ-819135 still has a smaller stiffness than ALJ-819127 after 10 cycles.

A small *C*_a_ indicates that the stiffness increase is small during the cyclic loading. Therefore, the coefficient *C*_a_ shows a positive correlation with the loading capacity of the WRI devices, while it shows A negative correlation with the preload and cyclic load amplitudes.

## 5. Conclusions

In this study, five WRI specimens were tested to obtain the hysteresis curves and the axial stiffness. The mechanical behaviors of the WRI specimens under repeated large loads were obtained with various loading amplitudes and velocities. Then, the specimens were loaded under small cyclic amplitudes and specified preloads to obtain the mechanical properties. Based on the test results and the corresponding analysis, the following conclusions are drawn:

(1) The WRI specimen shows stable mechanical behavior under various cyclic load amplitudes, and the hysteresis curves are independent of the loading speed. In addition, the hysteresis curves show little changes under the alternate compression and tension loads.
(2) The test results show that the stiffness of the WRI specimen follows the logarithmic law with the cycle number under various loading conditions. Particularly, the stiffness of the specimen increases by about 10–30% after 50 cycles.
(3) The initial stiffness *K*_a_ decreases linearly with the preloads, while it is quadratically related to the cyclic load. The coefficient *C*_a_ shows a positive correlation with the loading capacity of the WRI devices, while it shows a negative correlation with the preload and cyclic load amplitude.

## Figures and Tables

**Table 1:** Mechanical properties of the WRI product (from the manufacturer).

Type	Name	Maximum Static Load (kN)	Maximum Deformation (mm)	Height (mm)	Length (mm)	Width (mm)	Diameter (mm)
ALJ-819135	S1	2.80	74.0	135	267	178	12.7
ALJ-8110133	S2	12.28	44.8	133	368	140	22.2
ALJ-819127	S3	3.18	67.0	127	267	165	15.9
ALJ-819109	S4	4.204	49.5	109	267	135	15.9
ALJ-81989	S5	6.45	32.0	89	267	102	15.9

**Table 2:** Test scenarios of the specimens.

Name	Specimen	Preloads	Loads	Loading Speed (mm/min)	Cycles Number
T1-S2-D1	ALJ-8110133	0	2.0 mm	0.5	40
T1-S2-D2		0	2.0 mm	1.0	50
T1-S2-D3		0	2.0 mm	2.0	30
T1-S2-D4		0	5.0 mm	5.0	60
T1-S2-D5		0	10.0 mm	5.0	35
T1-S2-D6		0	10.0 mm	10.0	120
T1-S2-D7		0	12.5 mm		10
T1-S2-D8		0	15.0 mm		60
T1-S2-D9		0	20.0 mm		85
T2-S1-F1	ALJ-819135	50% STC	20% STC		50
T2-S1-F2		50% STC	20% STC		
T2-S1-F3		50% STC	20% STC		
T2-S1-F4		50% STC	20% STC		
T2-S1-F5		50% STC	20% STC		
T2-S1-D6		50% STC	0.23 mm		
T3-S1-F1		20% STC	20% STC		
T3-S1-F2		35% STC	20% STC		
T3-S1-F3		50% STC	20% STC		
T3-S1-F4		65% STC	20% STC		
T3-S1-F5		80% STC	20% STC		
T3-S1-F6		50% STC	20% STC		
T3-S1-F7		50% STC	40% STC		
T3-S1-F8		50% STC	60% STC		
T3-S1-F9		50% STC	80% STC		
T4-S2-F1	ALJ-8110133	50% STC	20% STC		
T4-S3-F2	ALJ-819127	50% STC	20% STC		
T4-S4-F3	ALJ-819109	50% STC	20% STC		
T4-S5-F4	ALJ-81989	50% STC	20% STC		

## Data Availability

The original contributions presented in the study are included in the article, further inquiries can be directed to the corresponding author.
